# Folic acid phenotype (FAP) is a superior biomarker predicting response to pemetrexed-based chemotherapy in malignant pleural mesothelioma

**DOI:** 10.18632/oncotarget.16398

**Published:** 2017-03-21

**Authors:** Fabian Dominik Mairinger, Claudia Vollbrecht, Elena Flom, Daniel Christian Christoph, Kurt-Werner Schmid, Jens Kollmeier, Helmut Hans Popper, Thomas Mairinger, Robert Fred Henry Walter

**Affiliations:** ^1^ Institute of Pathology, University Hospital Essen, University of Duisburg-Essen, Essen, Germany; ^2^ Institute of Pathology, Division of Molecular Pathology, Charité, Berlin, Germany; ^3^ German Cancer Consortium (DKTK), Germany; ^4^ German Cancer Research Center (DKFZ), Heidelberg, Germany; ^5^ Department of Medical Oncology, West German Cancer Centre, University Hospital Essen, University of Duisburg-Essen, Essen, Germany; ^6^ Department of Pneumology, Helios Klinikum Emil von Behring, Berlin, Germany; ^7^ Department of Pathology, Division of Molecular Lung- and Pleurapathology, Medical University of Graz, Graz, Austria; ^8^ Department of Pathology, Helios Klinikum Emil von Behring, Berlin, Germany; ^9^ Ruhrlandklinik, West German Lung Centre, University Hospital Essen, University of Duisburg-Essen, Essen, Germany

**Keywords:** pleural mesothelioma, pemetrexed, thymidylate synthethase, folylpolyglutamate synthase, personalized therapy

## Abstract

**Background:**

Malignant pleural mesothelioma (MPM) is a rare tumor linked to a dismal prognosis. Even the most effective chemotherapeutical regime of pemetrexed combined with cisplatin leads to a remission-rate of only about 40%. The reasons for the rather poor efficacy remain largely unknown.

**Results:**

Phenotypes were significantly associated with progression (p=0.0279) and remission (p=0.0262). Cox-regression revealed significant associations between *SLC19A1*/*TYMS*-ratio (p=0.0076) as well as *FPGS*/*TYMS*-ratio (p=0.0026) and OS. For differentiation by risk-groups, COXPH identified a strong correlation (p=0.0008).

**Methods:**

56 MPM specimens from patients treated with pemetrexed were used for qPCR analysis. Phenotypes and risk groups were defined by their expression levels of members of the folic acid metabolism and correlated to survival and objective response.

**Conclusion:**

Our results indicate that the balance between folic acid uptake, activation and metabolism plays a crucial role in response to pemetrexed-based chemotherapy and the prognosis of MPM patients. Implementing this marker profile in MPM stratification may help to individualize MPM-therapy more efficiently.

## INTRODUCTION

Malignant pleural mesothelioma (MPM) is a rare, biologically highly aggressive tumor leading to a dismal prognosis [1, 2]. Standard MPM therapy is still not optimal, and decisions for surgery, radiotherapy or multimodal procedures are made case-by-case. Mostly, a palliative treatment approach remains the only choice [3, 4]. In clinical practice, the antifolate pemetrexed, as the only FDA-approved therapeutic for MPM, is used in combination with platin compounds [5–9].

**Figure 1:**
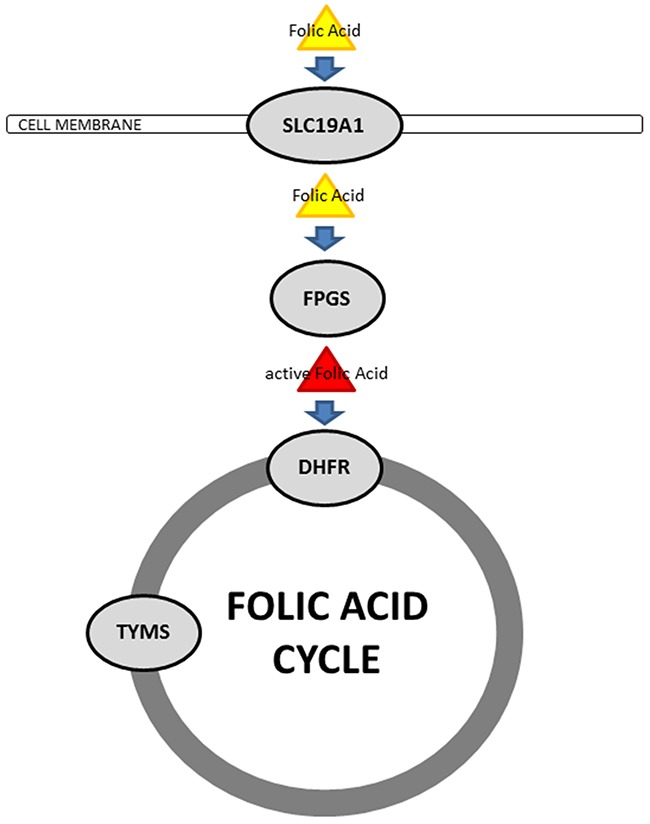
Schematic illustration of cellular folic acid metabolism In MPM, folic acid and antifolates get taken up into the cell by the reduced folate carrier (*SLC19A1*). The intracellular activation of antifolates is performed by polyglutamylation, catalyzed by the folylpolyglutamate synthase (*FPGS*). These active folates get implemented to the folic acid cycle by the dihydrofolate reductase (*DHFR*) and further on furnished to different cellular processes. Catalytic efficacy of the folic acid cycle is set by the thymidylate synthethase (*TYMS*).

Several studies have shown the efficacy of the evaluation of intra-tumoral expression of thymidylate synthethase *(TYMS)* mRNA for prediction of multitargeted antifolate therapy response in patients with breast cancer [10], colorectal cancer [11], head and neck cancer [12], pancreatic cancer [13] and NSCLC [14, 15]. However, these associations are discussed controversially [16–19]. Thus, it is worth to consider another approach focusing on intracellular transport and activation of antifolates [18, 19]. Generally, folic acid and antifolates uptake into the cell is carried out either by reduced folate carrier (*SLC19A1*) or by folate receptor-1 (*FOLR1*), whereas MPM predominantly use *SLC19A1* [20]. The polyglutamylation of antifolates and their activation is catalyzed by the folylpolyglutamate synthase (*FPGS*) [21] (Figure 1).

As none of these single markers could *de facto* improve patients’ outcome suffering by MPM until now, we hypothesize that the balance between cellular antifolate uptake, activation and metabolism might be a potential biomarker. Therefore, we analyzed this equilibrium by defining a folic acid phenotypes (FAP) to rank patients by their probability of a response to pemetrexed-based chemotherapy.

## RESULTS

CART analysis determined 0.43 as cut-off for *SLC19A1* and 1.89 as cut-off for *TYMS* gene expression levels regarding FAP1. For FAP2, CART analysis rendered 0.34 as cut-off for *FPGS* and 0.90 as cut-off for *TYMS*. For risk-group determination, thresholds were calculated with >1.25 for the low-risk group, 0.6-1.25 for the intermediate-risk group and <0.6 for the high-risk group.

**Figure 2:**
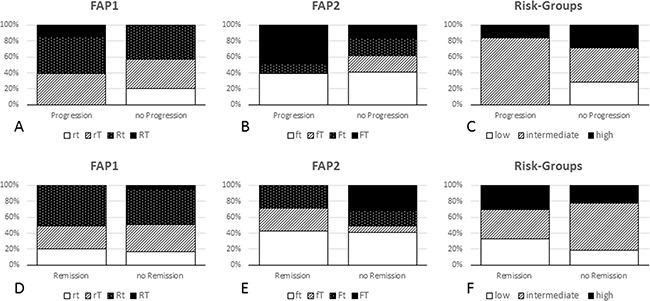
FAP1, FAP2 and risk groups in correlation with objective response (modRECIST) The upper line shows data for progression and the lower line depicts data for remission. For FAP1 **(A** and **D)**, the rt-phenotype was found in patients without initial radiologic tumor progression only and was more frequently seen remissive samples. In contrast, the RT-phenotype appears in progressive samples only. For FAP2 **(B** and **E)**, the FT-phenotype was prominent in progressive cases and absent in remissive cases. Of note, the fT-phenotype exclusively associates with non-progressive disease and was found in one-fourth of remissive cases. Considering the defined risk-groups **(C** and **F)**, the low risk-group never showed progression under pemetrexed-based chemotherapy.

FAP1 was significantly associated with objective tumor response including progression (p=0.0279) and remission (p=0.0262) under pemetrexed-based chemotherapy. The rt-phenotype was only found in tumors with no progression, similarly the RT-phenotype was exclusively observed in progressive cancers. Additionally, the RT-phenotype was not detected in samples with remission (Figure 2). Although, FAP2 not just yet passed statistical significance when correlated to remission only (p=0.0595), it is striking that the FT-phenotype is present in about 40% of all tumors without remission, but absent in tumors showing remission. Of note, the fT-phenotype appeared exclusively in non-progressive patients (Figure 2).

Tumors in the low-risk group did not show progression, but were present in more than 30% of all tumors with remission (Figure 2).

The Cox-regression revealed significant association between high *SLC19A1*/*TYMS*-ratios and prolonged OS (p=0.0076, HR: 1.48) as well as high *FPGS*/*TYMS*-ratios and prolonged OS (p=0.0026, HR: 2.18). Especially, the 3-year survival rate differs strongly between these groups (*SLC19A1*/*TYMS*-high: >40% vs. *SLC19A1*/*TYMS*-low: <15%; *FPGS*/*TYMS*-high: >50% vs. *FPGS*/*TYMS*-low <15%).

Risk-groups correlated with OS (p=0.0008, HR: 5.34), although the low-risk group shows a prolonged survival only.

**Figure 3:**
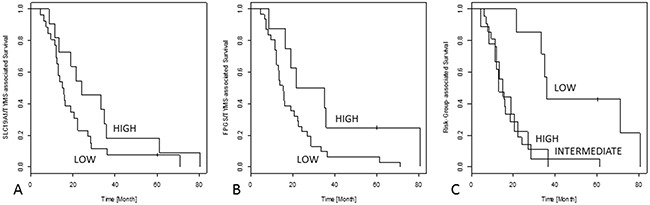
Kaplan-Meier curves for the calculated ratios and risk groups are shown In **(A)**, the *SLC19A1*/*TYMS* ratio is shown, leading to prolonged 3-year survival if the receptor activation is prevailing. Furthermore, a shift towards antifolate activation demonstrated by the *FPGS*/*TYMS* ratio leads to a survival benefit **(B)**. In **(C)**, the low-risk group gains a better outcome than the other two risk groups.

Survival analysis is illustrated in Figure 3.

## DISCUSSION

The value of folic acid pathway members as a marker for predicting disease response to antifolate therapy remains controversial [9–19, 22–31]. These controversially discussed approaches focused on single members of the folic acid metabolism only. In our previous investigations, we focused on different enzymes within the thymidine cycle and could not demonstrate a significant correlation to these enzymes [18, 19]: *FPGS* itself (p=0.011) as well as *SLC19A1* (p=0.0088) reached statistical significance, but especially for *SLC19A1* the group of identified well-responders was very low (4.8%). *TYMS* did not reach statistical significance at all (p=0.6611). Also other studies show similar significances and similar survival-benefits, *TYMS* itself is discussed controversially [24, 27]. In addition, a clinical investigation into NSAIDs, which might influence retention and thus prolonged bioavailability of pemetrexed, did also not show a correlation with patients’ response [19]. The results of a recent study of our group on neuroendocrine lung tumors [32] suggests that balance of cellular folic acid uptake, activation and metabolism is a driving factor of malignancy in these tumors. Therefore, we further proposed that the clue for multi-targeted antifolate resistance underlies the same mechanism, characterized by the two FAP1 and FAP2 named phenotypes. Each split four different stages of equilibrium between uptake and metabolism (FAP1) and activation and metabolism (FAP2). In addition, concerning all three levels of cellular folic-acid processing at once, we defined risk-groups differentiating long-time responders as well as primarily resistant patients.

Through combining those markers calculating ratios to display their interrelation, strong significances to OS were found (*FPGS*/*TYMS*: p=0.0026; *SLC19A1*/*TYMS*: p=0.0076). Of note, the *FPGS*/*TYMS* ratio identifies a subgroup of responders (18.6%) with a 3-year survival of >50%. Similarly, the *SLC19A1*/*TYMS* ratio defines a subgroup of 26.8% with a 3-year survival rate of >40%. In addition, FAP1 as a comprehensive parameter based on the *SLC19A1*/*TYMS* ratio, reached statistical significance for each progression and remission. Especially, the RT-phenotype was found in progressive tumors only; contrarily, the rt-phenotype was found in non-progressive and in a superior portion of remissive cancers (20%) only. Even for FAP2 based on the *FPGS*/*TYMS* ratio, fT phenotype could be found in tumors showing no progression under pemetrexed-based chemotherapy only. In addition, the FT-phenotype was present in half of all progressive tumors, but absent in remissive cases. and can therefore support clinical decision making.

Most impressively, stratification by risk-groups reached statistical significance (p=0.0008) and identified a low-risk subgroup of well-responders (17.1% of the investigated patients) with a 3-year survival rate of approximately 80% and a five-year survival rate of still >40%. Interestingly, the high- and intermediate-risk groups did not gain any benefit, indicating that at a certain point revert of this equilibrium do not generate a further beneficial graduation.

Of note, not only remissive but also patients with stable disease during pemetrexed treatment benefit from this therapy. We tried to indicate this interrelation by defining the term “progression” as PD vs. SD/PR/CR. To our understanding, the phenotypes correlated with progression such as RT and rt, respectively (Figure 2A - FAP1, only present/absent in progressive tumors), or fT (Figure 2B - FAP2, only present in non-progressive tumors) are diagnostically more relevant/applicable than phenotypes exclusively present in non-remissive tumors such as FT (Figure 2E – FAP2). Concordantly, the low-risk group as best marker for outcome prediction in this study appears exclusively in non-progressive tumors (Figure 2C) but shows just a slight over-representation in remissive compared to stable tumors (Figure 2F).

On the other hand, also a real reduction of the tumor mass (remission) indicating a prepotent induction of apoptosis or necrosis compared to proliferation leads to interesting conclusions and a better understanding of tumors’ mechanisms to deal with the chemotherapeutics. For future development and further stratification of patients, this information may be helpful.

In conclusion, our results indicate that the balance between folic acid uptake, activation and utilization plays a crucial role for response to pemetrexed-based chemotherapy and prognosis in MPM. Therefore, it would be of great importance to validate both folic acid phenotypes and risk groups in larger prospective studies to confirm these results, improve and individualize MPM-therapy, saving pemetrexed non-responder patients from inefficient and side-effect loaded antifolate therapy.

## MATERIALS AND METHODS

### Patient samples

Formalin-fixed, paraffin-embedded (FFPE) samples from 56 patients with MPM were selected for the study as described previously [19]. The patients were recruited from the biobank of the Department of Pathology, Helios Klinikum Emil von Behring, Berlin (Germany). Patient samples were obtained between 2002 and 2009. Inclusion criteria were the availability of sufficient tumor material, a history of at least one cycle of pemetrexed containing chemotherapy and the case to be listed in the clinical registry for tumor response and survival. All specimens were collected prior to systemic treatment. For survival analysis, only tumors showing an epithelioid histology were included to overcome the problem of an inhomogeneous dataset, resulting in 44 patients included. Response data were evaluated centralized using the modified Response Evaluation Criteria in Solid Tumors (modRECIST) for assessment of response in MPM [33]. For a better stratification, remission was defined by complete response (CR) and partial response (PR) versus stable disease (SD) and progressive disease (PD). Likewise, progression was defined by CR and PR and SD versus PD. An overview of patient data is given in Table 1.

**Table 1:** Overview of clinical and histopathological parameters for each patient

Gender	Age (Years)	Survival (Months)	Histological Subtype	Surgery	Chemotherapy	Response (modRECIST)
M	70	67,43	E	Decortication	4 cycles Cisplatin/Pemetrexed 2nd line	SD
M	68	72,1	E	Decortication	5 cycles Cisplatin/Pemetrexed 2nd line	SD
M	57	36,43	E	Decortication	6 cycles Cisplatin/Pemetrexed 1st line	SD
M	66	28,9	E	Pleuropneumonectomy	6 cycles Carboplatin/Pemetrexed 2nd line	PD
F	77	34,77	E	Pleurodesis	3 cycles Carboplatin/Pemetrexed plus 2 cycles Pemetrexed monotherapy 1st line	SD
M	72	21,9	E	Decortication	4 cycles Carboplatin/Pemetrexed 1st line	SD
M	59	8,93	E	Pleurodesis	2 cycles Carbo/Pemetrexed 1st line	PD
M	62	7,27	E	Pleurodesis	1 cycle Carboplatin/Pemetrexed 1st line	NA
M	62	16,6	E	Decortication	4 cycles Platin/Pemetrexed 1st line	NA
M	76	33,93	E	Pleurodesis	6 cycles Carboplatin/Pemetrexed 1st line	SD
M	59	7,27	E	Decortication	6 cycles Cisplatin/Pemetrexed 1st line	SD
M	82	13,5	E	Pleurodesis	6 cycles Pemetrexed Monotherapy 1st line	SD
M	70	48,83	E	none	6 cycles Platin/Pemetrexed 1st line	PR
M	67	20,93	E	none	4 cycles Carboplatin/Pemetrexed 1st line	PD
M	62	35,47	E	Decortication	4 cycles Cisplatin/Pemetrexed 1st line	PR
M	49	16,1	E	Pleuropneumonectomy	4 cycles Cisplatin/Pemetrexed (adjuvant therapy)	NA
F	77	81,73	E	Decortication	4 cycles Carboplatin/Pemetrexed 1st line und 6 cycles 2nd line	PR
M	68	53,33	E	Decortication	4 cycles Cisplatin/Pemetrexed 1st line	SD
M	71	15,43	E	Pleurodesis	1 cycle Carboplatin/Pemetrexed 1st line	PD
M	67	16,23	B	Decortication	4 cycles Carbo/Pemetrexed 1st line	PD
M	65	9,67	E	Decortication	2 cycles Carbo/Pemetrexed 1st line	NA
M	67	19,13	E	Pleurodesis	6 cycles Carbo/Pemetrexed 1st line	SD
F	86	27,37	E	Pleurodesis	8 cycles Pemetrexed Mono 1st line	PR
M	69	15,87	E	Decortication	3 cycles Platin/Pemetrexed 1st line	PD
F	63	24,53	E	Decortication	6 cycles Carbo/Pemetrexed 1st line	PR
M	77	62,07	E	Decortication	6 cycles Platin/Pemetrexed 1st line	PR
F	73	48,9	E	Decortication	3 cycles Cisplatin/Pemetrexed 1st line	PD
M	69	16,1	E	Pleuropneumonectomy	4 cycles Cisplatin/Pemetrexed (adjuvant therapy)	NA
F	64	9,07	E	none	2 cycles Platin/Pemetrexed 1st line	PD
M	67	11,5	S	Decortication	6 cycles Cisplatin/Pemetrexed 1st line	SD
M	73	5,2	E	Pleurodesis	3 cycles Pemetrexed Mono 1st line	NA
M	66	12,33	E	Decortication	6 cycles Cisplatin/Pemetrexed 1st line	SD
M	67	8,53	S	Decortication	4 cycles Carbo/Pemetrexed 1st line	PD
F	alive	n.n.	E	Pleurodesis	6 cycles Cisplatin/Pemetrexed 1st Line	PR
M	76	22,50	B	Decortication	4 cycles Carboplatin/Pemetrexed 1st Line	PR
M	77	28,6	E	Decortication	4 cycles Cisplatin/Pemetrexed 1st Line	SD
M	71	18,77	E	Decortication	4 cycles Cisplatin/Pemetrexed 1st Line	SD
M	72	40,37	E	Decortication	4 cycles Carbo/Pemetrexed 1st Line	SD
F	54	10,1	E	Decortication	5 cycles Cisplatin/Pemetrexed 1st Line	PD
F	64	11,57	B	Decortication	4 cycles Cisplatin/Pemetrexed 1st Line	PD
M	81	6,33	B	Decortication	3 cycles Pemetrexed Mono 1st Line	PD
M	73	4,67	B	Decortication	1 cycle Carboplatin/Pemetrexed 1st Line	NA
M	69	8,13	B	Pleurodesis	4 cycles Cisplatin/Pemetrexed 1st Line	PD
M	75	15,37	E	Decortication	4 cycles Cisplatin/Pemetrexed 1st Line	PR
M	71	22,73	B	Decortication	6 cycles Cisplatin/Pemetrexed 1st Line	PR
M	61	25,63	E	Decortication	4 cycles Cisplatin/Pemetrexed 1st Line	SD
M	38	37,03	E	Decortication	6 cycles Cisplatin/Pemetrexed 1st Line	PR
M	79	19,27	E	Decortication	6 cycles Carbo/Pemetrexed 1st Line	SD
F	61	12,27	E	Decortication	5 cycles Cisplatin/Pemetrexed 1st Line	SD
M	74	29,03	E	Decortication	5 cycles Carbo/Pemetrexed 1st Line	SD
F	79	13,77	S	Decortication	6 cycles Carbo/Pemetrexed 1st Line	SD
F	64	4,03	E	Decortication	3 cycles Cisplatin/Pemetrexed 1st Line	PD
M	alive	n.n.	E	Decortication	6 cycles Carbo/Pemetrexed 1st Line	SD
M	71	18,13	E	Decortication	6 cycles Cisplatin/Pemetrexed 1st Line	SD
M	60	13,23	E	Decortication	6 cycles Cisplatin/Pemetrexed 1st Line	SD
M	66	12,27	E	Decortication	6 cycles Cisplatin/Pemetrexed 1st Line	PD

Patients gender, age at time of diagnosis, overall survival, histological subtype as well as therapeutic strategies and response rate are shown. Response data were evaluated using the modified Response Evaluation Criteria in Solid Tumors (modRECIST).

The investigation conforms the principles outlined in the Declaration of Helsinki, and all patient samples were anonymized. An informed consent of all patients is available.

### Gene expression analysis

RNA-Isolation was performed with the RNeasy FFPE kit from Qiagen (Venlo, Netherlands). Therefore, three to five section of each 20μm thickness were used and tumor areas were macrodissected to reach at least 80% of tumor cells. cDNA synthesis was performed with the iScript^®^ Select cDNA Synthesis Kit from BioRad^®^ (Hercules, CA, USA) using 2 μg RNA (200 ng/μl).

Relative cDNA quantification of *TYMS*, *SLC19A1* and *FPGS* was analyzed by the 2^-ΔCt^-method. For biological normalization purposes, gene expression values were normalized to reference gene expression in each sample. *ACTB* and *GAPDH* were selected as reference genes using the geNorm and NormFinder algorithm. Evaluation was carried out with commercial TaqMan^®^ Gene Expression-assays (Applied Biosystems^®^, Foster City, CA, USA; *TYMS*: Hs00426586_m1, *GAPDH*: Hs00266705_g1, *ACTB*: Hs01060665_g1, *SLC19A1*: Hs00953344_m1, *FPGS*: Hs00909430_m1). For the gene expression analysis in FFPE tissue, a set of primers with small amplicon size (<100bp) was used, in order to overcome the limits of RNA degradation [34]. qPCR and data analysis was performed on a Roche^®^ LightCycler^®^ 480 (Roche Applied Sciences, Penzberg, Germany).

Pipetting steps were conducted fully automated by a Hamilton^®^ pipetting robot (Reno, NV, USA). Each sample was measured in triplicate. The efficiency of all used assays was calculated by their standard curves using six different concentrations from a pool of all isolated RNAs. qPCR analysis was performed in concordance to the MIQE-guidelines [35, 36].

### Statistical analysis

All statistical analyses were calculated using the R i386 statistical programming environment (v3.2.3) [37]. Phenotypic sorting was performed by classifying samples as either having mRNA expression counts above the cut-off (depicted by capital letters e.g. R, F or T) or below the cut-off (depicted by lowercase letters e.g. r, f or t). Phenotypes were defined by the expression of *SLC19A1* and *TYMS* (FAP1; RT-Rt-rT-rt) and *FPGS* and *TYMS* (FAP2; FT-Ft-fT-ft), respectively.

Risk-groups were defined due to the ratio between *SLC19A1* and *FPGS* to *TYMS*.

All thresholds were determined by Classification and Regression Tree Algorithm (CART) using ANOVA regression model. Overall survival (OS) was calculated by the Kaplan-Meier method. Analyses of associations between gene expression and the overall survival (from time of diagnosis) was done by Cox-regression (COXPH-model), statistical significance was determined using Likelihood ratio test. Level of statistical significance was defined as p=0.05.
